# Co-Creation With TickiT: Designing and Evaluating a Clinical eHealth Platform for Youth

**DOI:** 10.2196/resprot.2865

**Published:** 2013-10-18

**Authors:** Sandy R Whitehouse, Pei-Yoong Lam, Ellen Balka, Shelagh McLellan, Mariana Deevska, Daniel Penn, Robert Issenman, Mary Paone

**Affiliations:** ^1^BC Children's HospitalDivision of Adolescent MedicineUniversity of British ColumbiaVancouver, BCCanada; ^2^Senior Scholar, Michael Smith Foundation for Health ResearchSchool of CommunicationSimon Fraser UniversityBurnaby, BCCanada; ^3^Umeå Institute of DesignUmeå UniversityUmeåSweden; ^4^Shift Health Paradigms Ltd.Toronto, ONCanada; ^5^McMaster Children's HospitalDepartment of PediatricsMcMaster UniversityHamilton, ONCanada

**Keywords:** adolescent, adolescent health services, youth, eHealth, information technology, health surveys, delivery of health care, communication, chronic illness, mobile technology, questionnaires

## Abstract

**Background:**

All youth are susceptible to mental health issues and engaging in risky behavior, and for youth with chronic health conditions, the consequences can be more significant than in their healthy peers. Standardized paper-based questionnaires are recommended by the American Academy of Pediatrics in community practice to screen for health risks. In hospitals, psychosocial screening is traditionally undertaken using the Home Education, Eating, Activities, Drugs, Depression, Sex, Safety (HEEADDSS) interview. However, time constraints and patient/provider discomfort reduce implementation. We report findings from an eHealth initiative undertaken to improve uptake of psychosocial screening among youth.

**Objective:**

Youth are sophisticated “technology natives.” Our objective was to leverage youth’s comfort with technology, creating a youth-friendly interactive mobile eHealth psychosocial screening tool, TickiT. Patients enter data into the mobile application prior to a clinician visit. Response data is recorded in a report, which generates alerts for clinicians, shifting the clinical focus from collecting information to focused management. Design goals included improving the patient experience, improving efficiency through electronic patient based data entry, and supporting the collection of aggregated data for research.

**Methods:**

This paper describes the iterative design and evaluation processes undertaken to develop TickiT including co-creation processes, and a pilot study utilizing mixed qualitative and quantitative methods. A collaborative industry/academic partnership engaged stakeholders (youth, health care providers, and administrators) in the co-creation development process. An independent descriptive study conducted in 2 Canadian pediatric teaching hospitals evaluated the feasibility of the platform in both inpatient and ambulatory clinical settings, evaluating both providers and patient responses to the platform.

**Results:**

The independent pilot feasibility study included 80 adolescents, 12-18 years, and 38 medical staff-residents, inpatient and outpatient pediatricians, and surgeons. Youth uptake was 99% (79/80), and survey completion 99% (78/79; 90 questions). Youth found it easy to understand (92%, 72/78), easy to use (92%, 72/78), and efficient (80%, 63/79 with completion rate < 10 minutes). Residents were most positive about the application and surgeons were least positive. All inpatient providers obtained new patient information.

**Conclusions:**

Co-creative design methodology with stakeholders was effective for informing design and development processes to leverage effective eHealth opportunities. Continuing stakeholder engagement has further fostered platform development. The platform has the potential to meet IHI Triple Aim goals. Clinical adaptation requires planning, training, and support for health care providers to adjust their practices.

## Introduction

### Overview

The Institute of Health Improvement (IHI) recommends that new health care strategies are designed to meet the Triple Aim goals. These goals are described as (1) improving the patient experience, (2) reducing or maintaining costs, and (3) improving health of the population. eHealth innovation, through harnessing both information technology (IT), communication technology, and layering capabilities, has the potential to meet the Triple Aim goals through “multitasking” by supporting efficient collection of patient information, distribution to providers and patients, and re-purposing for health research. eHealth commonly refers to health services and information delivered or enhanced through the Internet and related technologies. The “e”s in eHealth align with traditional medical practice in enhancing quality and evidence-based care, while providing the opportunity to achieve a number of other “e”s such as empowerment, efficiency, encouragement of new relationships between providers and patients, enabling information exchange, and extending the scope of health care [1].

While the face-to-face patient encounter remains a critical element of health care provision, a single eHealth intervention could improve the patient experience of the health care encounter, educate the patient, collect important clinical information, improve efficiency, and support aggregation of data, which in turn can support the development of evidence to inform health care interventions [1]. However, without good design and engagement of stakeholders, new tools can just as easily miss their targets [2]. Design strategies are important considerations in development when tools are created to bridge between different stakeholder groups. Simply integrating technology to existing practices does not ensure realization of the complete potential of the technology nor does it necessarily improve the existing practices. In health care, there are a variety of stakeholders who interact with the technology, who each come with their own expectations, priorities, and limitations. Youth are technology natives, health care providers (HCPs) are content driven but often fearful of new technology, while administrators and IT personnel are concerned with technical standards, safety, and cost. A successful tool needs to be accessible to multiple stakeholder groups, supporting each group’s constraints and requirements as well as addressing their various perspectives and priorities.

Adolescence (12-18 years) and the extended period of youth (14-24 years) are a developmental life phase in which the opportunities for good health are great and future patterns of adult health are established. Youth health is influenced by social role changes, shaped by social determinants and risk and protective factors that affect the uptake of health related behaviors [3]. It is also a time when lifestyle choices and risky behavior can lead to significant morbidity. Adolescents are more likely than adults to binge drink, smoke cigarettes, have casual sex partners, and engage in violent behavior [4].

Comprehensive psychosocial health screening is a fundamental component of adolescent health care. Screening provides an opportunity to assess progress through adolescence and identify strengths and areas of concern. This information is essential to direct health promotion interventions. Several health organizations have written policy recommendations to encourage widespread practice of screening to promote optimal physical, mental, and social health [5-7]. These recommendations include annual health screening of all adolescents in settings where youth interact with HCPs such as clinical outpatient or inpatient settings, public health, and school settings.

For general patient visits in a community setting, the American College of Pediatrics has developed paper-based Guidelines for Adolescent Preventive Services (GAPS) as part of their Brighter Futures initiative [8]. The 15-item survey focuses on risk behaviors, and has been shown to identify risk factors in youth attending community settings. In the hospital setting, psychosocial screening is undertaken by semi-structured interview using the acronym HEEADDSS (Home, Education, Eating, Activities, Drugs, Depression, Sexual Health, Safety) [9] as part of the admission procedure. The guided interview format moves progressively from less general topics to more sensitive issues. However, this method for universal inpatient or outpatient screening has proven unrealistic for a number of reasons. It is time consuming, taking on average 30 minutes per interview [10] and furthermore it requires skill, knowledge, and a comfort level by HCPs to address sensitive issues. Reviews of inpatient psychosocial screening in a pediatric inpatient setting determined documentation rates of only 50% [11], and as low as 19% in a surgical setting [12]. Despite the low uptake in a surgical ward, screening resulted in a 30% increase in referrals, highlighting the value of HEEADDSS in uncovering new health issues [12].

A paper-based self-administered questionnaire the Adolescent Screening Questionnaire (ASQ) [13] improved identification of risk factors and documentation in an inpatient setting. Additionally, the ASQ increased efficiency, taking approximately 10 minutes to complete. However uptake was inadequate as 25% of adolescents declined to participate. Those who did fill in the questionnaire commented on the quality of experience, noting the questions could have a more positive focus [13].

Electronic (computer) based surveys are increasingly used in clinical settings in the clinic waiting rooms, generating a risk report for the clinician [14]. Olson reported a PDA based tool used in a community general pediatric clinic for adolescents attending a health checkup that improved the adolescents’ perceptions of the visit. Specifically, it also improved the patients’ perception that the clinician had listened carefully to them, and reduced the number of questions that they would have liked to discuss but did not [15]. Improving communication, changing focus from collecting information and redirecting it to focus on already determined risk and protective factors improved compliance of the assessment [16]. Paperny et al found computer assisted delivery of preventive services during a patient checkup in pediatric community practice was preferred (over face to face interview) by patients and reduced costs by 75% [17].

Building on the positive results from previous studies which used varied computer-based survey delivery as a means to collect psychosocial information to support clinical care, we attempted to bring traditional recommended standards of health care (the need for comprehensive psychosocial screening for youth) closer to the youth health space by creating an eHealth platform that was engaging and intuitive, while meeting other stakeholder requirements.

In this paper, our purpose is to both to demonstrate how mixed methods can contribute to effective design that meets stakeholder needs, and to highlight what we learned from a pilot study undertaken after initial development. Work reported here contributes to discussions about challenges of conducting research to inform ongoing design. Finally, this case demonstrates how engagement with stakeholders during design necessarily influences development of eHealth innovation.

**Figure 1:**
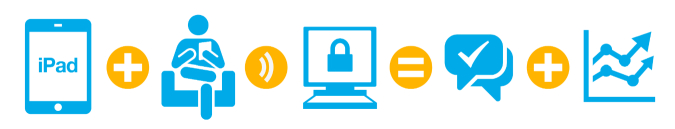
TickiT process banner.

Below, we outline the development of the platform, TickiT (Figure 1) from its inception as a mobile application, to a fully functional eHealth platform. The initial co-creation process was conducted with a collaborative industry and academic partnership with the Emily Carr University of Art and Design (ECUAD). After outlining the development process, we report on findings from an independent academic feasibility pilot study conducted in two Canadian teaching hospitals. Issues and challenges with platform development are discussed, as well as future directions for research.

### Background

Uptake of technology is dependent upon the quality of experience during implementation and use. Three stakeholder groups—adolescents, HCPs, and administrators—were identified as integral to implementation of the eHealth innovation described here.

Many health related survey tools have been directly transcribed from paper-based to electronic format with little consideration of the opportunities that migration from a paper-based to computer-based medium can afford. Willingness to experiment with question wording, graphic format, or survey content when moving from paper-based to computer mediated survey instruments may be constrained by challenges associated with norms and standards related to survey instrument validation. Validated surveys are constrained to maintain the original text if they are transcribed onto an electronic format. During our graphic design development, we found paper-based questions often appear long and inappropriate on an electronic interface. While the questions developed in the ASQ questionnaire module were previously validated, participants indicated that the tool could be more youth friendly in a study by Lam et al, which aimed to improve documentation of psychosocial screening in an inpatient setting [13]. While altering the wording or format of a validated instrument undermines validation, ample evidence suggests that altering survey instrument format when migrating content to a computer-mediated platform may support other affordances. For example, cognitive psychological research suggests that respondents encode questions into a mental representation as a starting point for question answering, providing graphic representation serves as a signal for memory retrieval and improves comprehension [18]. Graphics create the opportunity to reduce the literacy level, improving accessibility for a broader and younger population [19]. Additionally, youth engagement with technology has been associated with their comfort in disclosing personal information online, termed the “online disinhibition effect” [20].

Our project sought to address limitations in administration of youth health questionnaires by both moving data collection to an online platform. We chose to leverage new technology in creating TickiT, to improve the quality of youth experience and respond to youth preferences by altering the wording of questions from validated psychosocial screening instruments, while maintaining their meaning and enhancing the text with graphics on an interactive UI.

### The Co-Creation Method: Stage 1

We chose to utilize co-creation processes and methods with the goal of increasing patient engagement and simplifying HCP work, thereby improving patient/provider communication and experience while meeting regulatory requirements. Co-creation process shifts away from the traditional method of involving passive stakeholders during the latter phase of prototype testing towards viewing them as active contributors with knowledge and skills for co-creation during the ideation phase [21].

A design student from ECUAD undertook the initial co-creative research and subsequent preliminary development of TickiT with youth from the Youth Advisory Committee (12-20 years old, n=8; Co-Creative process: Development of psychosocial survey on an iPad platform, Ethics approval Emily Carr University of Art and Design). The youth participated in three 2-hour co-creation sessions. Subsequent co-creation sessions were held with adolescents in a high school (n=16) and a 1^st^ year design class at ECUAD (n=24). HCP staff (n=6) at British Columbia Children’s hospital (BCCH) were invited to participate in individual open-ended interviews about application development. Individual meetings reflected their time constraints and logistic challenges associated with arranging group sessions. The HCPs were professionally diverse and included a physician, 2 nurses, 2 social workers, and a developmental psychologist, each of whom could contribute a different viewpoint about the clinical encounter and inform the creation of developmentally appropriate content and context.

The patient visit trajectory (before appointment, registration, waiting room interaction with providers, after appointment, and back home) was presented to all the participants, youths, and HCPs through storyboarding which used the experience continuum design method [22] to evoke the varied contexts in which the tool might be used. The patient experience during a visit was considered using the domains of physical action, social interaction, and emotional reaction. Sessions were documented and participants were encouraged to write on material provided. All participants were offered the opportunity to send suggestions via email between and after the co-creation sessions.

The feedback from the co-creation sessions was used for the first functional prototype. This prototype platform was evaluated in an independent cognitive processing study to explore whether the type of icons are comprehensible and acceptable to ethnically diverse youth (E Saewyc, personal phone and email communication, July 27, 2013).

### Using the Platform in Practice: Stage 2

#### Overview

Once the application was functional, we needed to develop a better understanding of how it was being used, and what, if any issues, arose during implementation. A pilot study investigated the feasibility of using the platform in a hospital setting from both the youth and HCP perspectives. Lam et al [23] conducted a feasibility study as a 2^nd^ stage investigation of introducing standardized psychosocial screening in the hospital setting. In the 1^st^ stage of the study at BC Children’s Hospital, a chart review had determined that psychosocial screening was documented in 47% of the medical charts. Introduction of a standardized paper-based tool, the ASQ, had improved documentation, but 25% (10/40 invited to participate) of the youth refused to participate in completing the questionnaire and two youth suggested the questions could be more positive [13]. This low uptake rate was considered unacceptable, and a more youth friendly solution was sought as a means of improving compliance with the use of the psychosocial screening tool.

#### Goals

The goals of the 2^nd^ stage of the study were to determine if uptake of administration of psychosocial screening was improved by moving from a paper-based to tablet based administration of the psychosocial screening platform, to describe the youth and provider experience with using the user interface (UI) and questions both in the inpatient medical and surgical setting as well as the outpatient ambulatory care setting and to evaluate the efficiency of the eHealth platform. Efficiency was determined by time taken for the youth to complete the questionnaire as compared to standard provider/patient times for completing a HEADDSS interview [10]. Detailed information regarding the security features of the tool was provided in the ethics submission.

## Methods

### Stage 1

#### Co-Creation Sessions: Stakeholder Domains

##### Overview

Specific co-creation activities and processes undertaken to insure that application development responded to the needs of our three target stakeholder groups are outlined below.

##### Co-Creation Method: Youth

Many youth are disengaged from health care and uncomfortable in a clinical setting. Our primary consideration in the design of the UI was a Youth friendly approach [24-26]. Challenges related to process of engagement—uptake, engagement, efficiency, and confidentiality—were balanced with ensuring that the content was developmentally appropriate, comprehensible, and that the youth felt comfortable with how sensitive questions were being asked.

In the first group co-creation session, the youth brainstormed about the concept of psychosocial screening, in an attempt to determine strategies that could be followed to align their ideas with those of health professionals. The ASQ was used as a guiding text template for the survey as it was initially developed for use in a hospital setting [13]. Further, the ASQ used the categories derived from the HEADDSS assessment as the framework for sequencing the questions.

Focus groups were set up to discuss youth perception of content and language from the ASQ questionnaire and to suggest alterations to questions which would improve clarity, comprehension, engagement, and comfort [27]. As a result of youth co-creation input, the TickiT UI was initially implemented on paper, used 9 question categories (Home, Education, Eating, Activities, Depression [which at the suggestion of youth co-creators was changed to Emotions], Drugs, Safety, and Sex).

**Figure 2:**
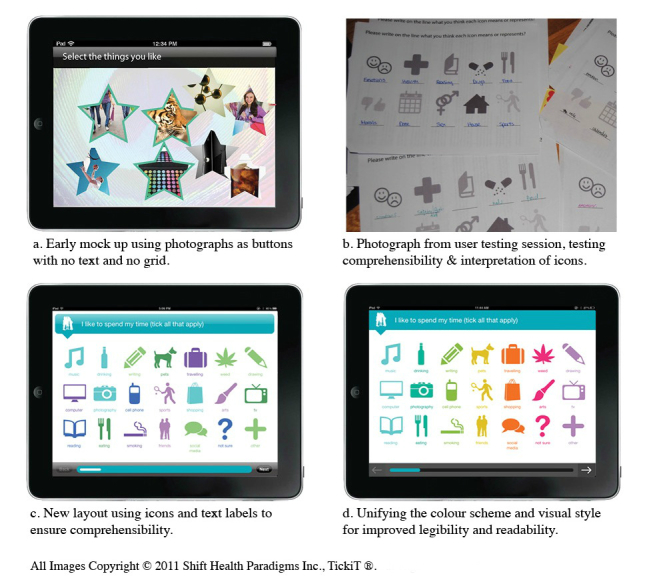
The Co-Creation process.

In subsequent sessions youth were provided with paper PDF copies of a version of questions reflecting revisions suggested from their previous feedback sessions (Figure 2). These were presented on color templates with icons for responses. Session participants were asked to interact with the interface, provide comments on the copies, and discuss their feelings about the UI. Feedback at this stage was sought specifically about the colors, size and readability of the text, comprehension of the questions, the youth’s sense of identification with the icons, and the mechanisms for answering questions. In the design development it was critical to ensure that the graphic elements and gestural interactions were universally understood and people with limited fine motor skills, for example, youth with neuromuscular conditions, could control the mobile device.

Youth were encouraged to discuss their attitudes towards the UI overall. They were asked their opinions regarding technical and feasibility issues of implementing the platform. This information was grouped using the subscales of Computerized Lifestyle Assessment Scale (CLAS) multidimensional computer survey evaluation [14] and included perceived (1) benefits, (2) concerns regarding privacy (consent, confidentiality, personal identifiers, data storage, connection with electronic health record), (3) interaction barriers (such as potential interference in their interaction with the physician), and (4) general interest. Responses were categorized using the CLAS themes, and youth commented on the relevance of the responses at the final co-creation session.

##### Co-Creation Method: Health Care Providers

An initial priority was to understand from HCP experts the requirements of developmentally appropriate care, which includes self-advocacy, lifestyle and risk, independent behaviors, sexual health, social supports, and educational, vocational, and financial planning.

The HCP goals for the tool included facilitating discussion, collecting information, generating a patient profile that could be uploaded into the electronic health record (EHR), a mechanism to collect aggregated anonymized data and a mechanism to track a patient’s progress when they used the survey on a subsequent occasion. Process issues for HCPs included time constraints, administration of the tool (who would administer it and how it would alter the workflow of clinical interactions), access to results, prioritizing information, data collection, and support for technical problems. Many were concerned about their lack of familiarity with technology.

Content was a high priority for HCPs. They recommended use of the HEEADDSS format, adopted such that it progressed from less personal to more personal questions. They were interested in obtaining the maximum amount of information depending on their special interests. For example, one participant commented that “I would like to have a more complete set of questions for my eating disorder clinic.” The HCPs were concerned that the quotes, which demarcated “chapters” of the questionnaire, endorsed healthy behaviors and were relevant – for example one HCP commented, “change the quote so that it is more appropriate for people with eating disorders.”

The HCPs made recommendations regarding report format, emphasizing simplicity, easy access, and attachment to an EHR. They confirmed the value of alerts on reports that reflected risk (red) and protective (green) factors and issues for concern (orange). The HCPs were keen to add other surveys onto the TickiT format. They prioritized future research validation and evidence of effectiveness.

##### Co-Creation Method: Institutional Requirements on Security and Regulation

Introducing new tools and technology into health care institutions require approval from administrative and IT departments. We undertook a detailed literature review and engaged security experts to ensure the system architecture and company policies complied with regulatory standards.

The management and implementation of an eHealth tool in Canada are governed by the provincial privacy legislation that is in place in each jurisdiction in which it does business, and by the federal Personal Information Protection and Electronic Documents Act (PIPEDA) [28,29]. Consideration of both provincial and federal jurisdictional laws were important as our first implementation sites were British Columbia (BC Children’s Hospital) and Ontario (McMaster University). Hence we had to meet various provincial regulations early in the development process of our software.

Privacy and security fall under 3 categories: organizational privacy, solution privacy, and risk analysis. Organizational privacy requires developing a comprehensive privacy program consisting of appointment of a Chief Privacy Officer, establishment of corporate privacy and information security policies, agreements with health organizations that address privacy roles and responsibilities, privacy training, monitoring and audit of all system activity, access to Personal Health Information (PHI) and implementing a breach management protocol**.** Solution privacy features relate to architectural design of the software and include features such as capturing consent, audit logging, secure storage of records, role based access control, end-user authentication, secure transmission of PHI over the Internet and ensuring PHI is not stored on tablets or end-user workstations. The privacy risk analysis uses risk mapping tools and criteria to evaluate risk, and analyze threat agents that might compromise PHI in some way. Privacy risk analysis also assesses threat agent motivation and capability, and identifies current safeguards in place as well as known vulnerabilities [28,29].

### Stage 2

Two Canadian hospitals participated in this stage of the study: McMaster Children’s Hospital and BCCH. The physicians involved in this study were exposed to the platform for the first time as a completed product and not aware of any previous developmental research with the platform. After obtaining both physician and youth consent (Behavioral Research Board Ethics approval, BC Children’s Hospital, and McMaster Children’s Hospital), 80 patients aged 12-18 years were invited to participate. In each of the clinical settings, every youth who met the eligibility criteria (age range and ability to read English), was consecutively approached for recruitment. No incentive to participate was provided. Inpatients were recruited from the BC Children’s Hospital Clinical teaching unit medical ward (n=15), a surgical ward (n=15), and ambulatory clinics including a cystic fibrosis clinic (n=15) and a youth health clinic (n=15). All the patients from McMaster were recruited from a gastroenterology clinic (n=20). One inpatient refused to participate (with the reason of being too sick) and one ambulatory patient did not complete the survey, resulting in 78 completed surveys and youth evaluations of the tool. Thirty-eight physicians who were caring for the youth patient participants in a Clinical Teaching Unit assessed the platform. This group included 13 staff physicians (3 inpatient medical, 2 surgical, 8 ambulatory) and 25 resident trainees who were working under the staff physicians. After consenting both patients and staff, the patients filled out the survey independently, either in their hospital room or in the ambulatory care waiting room. Thirty minutes later the research nurse collected the device, and the patients completed a paper-based evaluation of the UI and questions. The survey was presented as a series of short questions with a 5 point Likert scale and a small section at the end for further comments. The questions were the same for the youth from both centers, although the question regarding feeling comfortable with the survey questions was omitted from the McMaster cohort, due to a technical error. The research nurse then collected the report and provided the report with a paper-based survey evaluation of the report and questions to the available physician staff that was caring for the patient. If this was a trainee, another evaluation survey was provided for the responsible attending staff physician to complete as well. The provider evaluation survey was similar in format to the youth survey with Likert scale and a place for comments. The paper-based evaluation surveys were transcribed into Excel and uploaded to RedCAP for analysis. The patient questionnaire asked about comfort with the questions asked, comprehensibility and ease of use of the platform, and time taken to complete the survey. The provider questionnaire asked about the format of the report, the usefulness of the platform whether new information was obtained, and their comfort with receiving this information.

## Results

### Stage 1

#### Youth From Youth Co-Creation

The youth participants gave an overall very positive response regarding the concept of the tool. Some were concerned about privacy, and it was suggested that identifiers be limited to a number, age, and gender [30]. Many youth participants had suggestions regarding specific design constructs. For example they recommended graphic design be gender neutral with positive images and a colorful interface, and “different from school,” implying that it should not look like a test or school report. They suggested that many of the ASQ questions and the categorical headings they were grouped under had negative implications (eg, they pointed out that the category heading “Depression” is not neutral, and so this heading was subsequently changed to “Emotions”). They identified problems with the text, simplified questions and commented that it was more inviting to respond to text in the first person than the second person (eg, “I am male/female” instead of “Are you male/female?”). The youth identified potential judgmental icons (eg, the use of a check mark or “x” vs “yes/no”). They felt that “yes/no” was less subjective than a tick or cross that had positive or negative implications, reminiscent of a school marking system. The sexual health questions were the most controversial, and many found the question, “Which sex do you most identify with?” intimidating.

**Figure 3:**
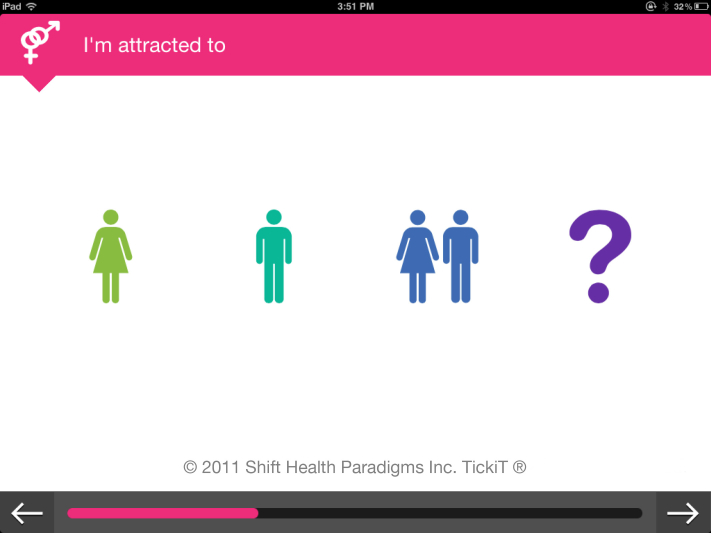
TickiT screenshot.

**Figure 4:**
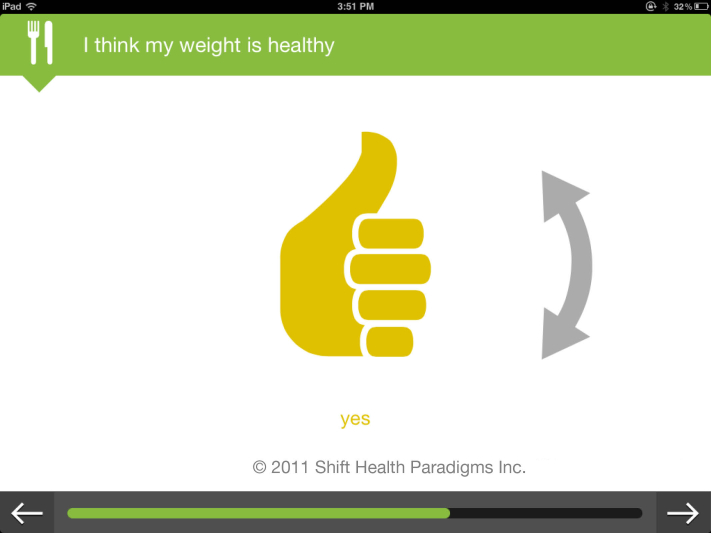
TickiT screenshot.

The youth were also concerned with technical issues. For example, one participant noted that “(we) need a way to select multiple icons.” They found the graphic format familiar. One commented that it “looks very apple-y” (Figures 3 and 4).

#### HCP Co-Creation

The flexibility of the UI platform architecture enabled us to meet many of the HCPs content requests, without making the survey cumbersome or seem overwhelming. The templates used made provision for detailed responses. For example, scales produce more variance than a simple True/False format and involve sliding a button. On the UI, Likert scale responses were made on a 4-point Likert-type scale (such as False, Mostly False, Mostly True, and True) rather than the more commonly used 5 point scales as it allowed us to avoid a tendency for youth to select a neutral response [31].

The survey was broken into sections, so that initial responses to questions could direct the survey in a variety of directions. This addressed the HCP concern of delving more deeply with further directed questions when indicated. As the platform was being used in a hospital setting with adolescents with a variety of physical and cognitive issues. Gestural and interactive responses had to be easy to use for adolescents with motor difficulties, and text had to be large enough to accommodate poor visual acuity. Language was kept at a Grade 4 literacy level.

The youths’ responses were accessed on a password protected website in the form of reports. Reports needed to be very simple to use, responding to many HCPs general anxiety with technology. The topic headings in the reports used the standardized format of the HEADDSSS assessment irrespective of the manner in which they were filled out as this was familiar to providers. The colored flagging system was enhanced with graphics for users with access only to black and white printers. Another feature of the website was the ability to download aggregated data for uploading into a database.

#### Security and Regulatory Domains: Tool to Platform

While we had originally conceived the development of a relatively simple eHealth tool, the security and regulatory requirements for data management necessitated considerable architectural design for data protection and auditing purposes, and ultimately supported evolution of TickiT from a survey tool to a data collection and management platform. For example, multiple levels of administrative permissions were built into the software to allow for multiple levels of administration, from changing the number of digits for the ID number, generating different user roles with varying levels of permissions, password management and restrictive security templates. The platform was allowed for more flexibility to meet different jurisdictional mandates, and provide a framework for further expansion and broader functionality in the future. Rigorous documentation of the platform’s security features together with policies and procedures to protect personal health information were required to ensure compliance with jurisdictional legislation on the management of personal health information, and for institutional ethics approval for the research studies. The program was designed to work with Internet Explorer 5, reflecting that many hospitals use older Web servers.

### Stage 2

#### Youth

**Figure 5:**
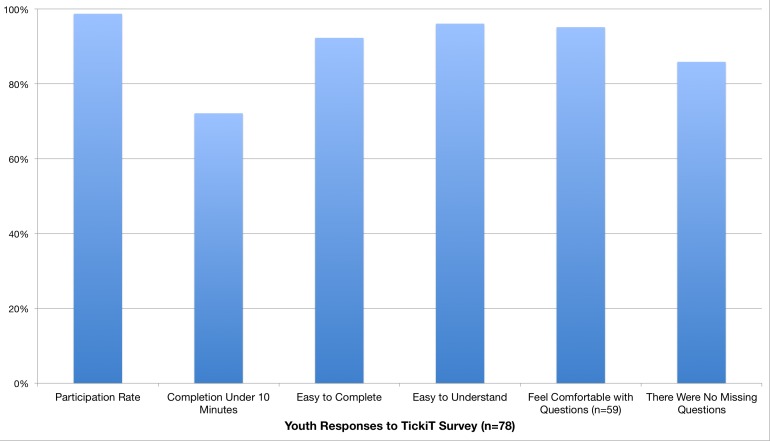
Youth perception of TickiT.

The youth participants were 56% (45/80) male ranging from 14-18 years old. Ninety-nine percent (79/80) of the youth agreed to participate. The single youth who declined was an inpatient that stated he felt too unwell to participate. Of the 79 youth who agreed to participate, 99% (78/79) completed all the questions. The number of questions ranged from 56-90 depending on the responses provided. Youth commented about their experience with the tool on a paper-based survey provided after they completed the questions. Eighty percent (63/79) of the youth reported they completed the questionnaire in less than 10 minutes and all completed it in less than 15 minutes. With respect to the user experience with the interface, 92% (72/78) of the youth reported the survey tool was easy or very easy to use. As far as content of the questions, 92% (72/78) found the questions easy or very easy to understand. While 91% (71/78) of the youth felt comfortable or very comfortable with the questions asked, 9% (7/78) were neutral and none of the youth reported that they felt uncomfortable with the content of survey questions (Figure 5). Youth were asked if they felt there were questions missing and were provided an opportunity to suggest questions to be added to the survey. One youth commented there could be more depth on some topics, but did not offer specific examples.

#### Health Care Provider

**Figure 6:**
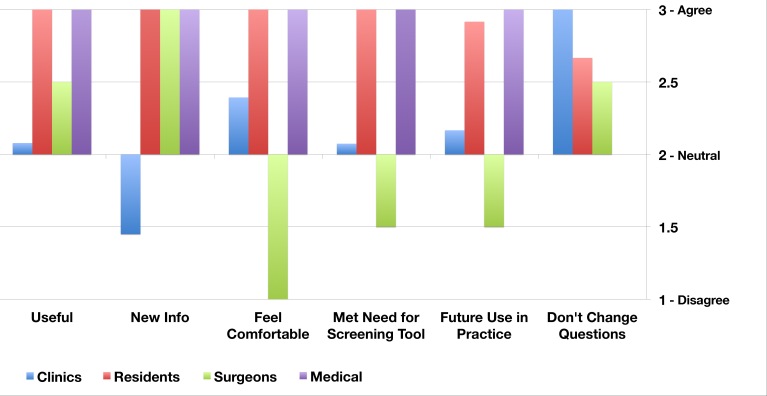
Health care provider perception of TickiT.

The HCPs responses varied between different professional groups, but were internally consistent. The resident trainees (n=25) gave universally positive evaluations of the platform. They found the information to be useful (25/25,100%), it met their needs as a screening tool (25/25,100%), and they felt that it had an acceptable report format (25/25,100%). Most residents (23/25, 92%) indicated that they would use TickiT in their future practice if it were available. All inpatient HCPs (pediatricians: n=3; surgeons: n=2) indicated that the reports provided new information about their patients. All the pediatricians felt comfortable with the survey platform (100%) and indicated that they would like to use TickiT in the future. However the two surgeons did not feel comfortable with the content of the reports, and commented that they did not have the skill set to manage the issues raised. They noted that they did not regularly obtain information gathered through the tool in their clinical practice. In the outpatient setting, the survey tool was given to follow-up patients with chronic health conditions who were receiving long-term medical care under a pediatric specialist (n=8). In this setting many of the HCPs (90%) did not receive new information, and most (85%) felt neutral about using the platform in the future. A couple (n=2) commented that they collected this information through other means. Despite their ambivalence, none of the physicians in any of the clinical settings recommended that any of the questions be changed. A summary of findings from these questionnaires is provided in Figure 6.

## Discussion

### Principal Findings

Here we have outlined the co-creation processes used during the development of an eHealth application which was initially conceived as a computer mediated means of conducting psychosocial screening of youth in varied health care settings and improving patient and HCP experiences. Over time, it emerged into a patient friendly data collection and management platform that can provide a report to HCPs, and securely captures screening data for subsequent use for research purposes.

We have presented findings from two pilot studies performed to evaluate the degree to which to the tool could increase compliance with psychosocial screening and to identify issues arising in participation by HCPs in varied settings. The co-creative process was used to develop the youth UI as youths are discerning users of technology who have high expectations, many of which are not being met by developers [32].

Youth participation, acceptance, and comfort using the tool in the Canadian hospitals study were exceptional, demonstrating the importance of being responsive to user suggestions from inception. Our findings suggest that this tool not only meets the standards recommended by health organizations for psychosocial screening but has the ability to improve health outcomes by easily providing the HCPs with valuable information [25,33]. However the relationship of the use of the platform and improved health outcomes can only be further evaluated with increased uptake of the tool and subsequent evaluation. The design strategy for development was effective for the HCP interface. The HCPs universally found the reporting interface acceptable, which indicated the information was readily available and easy to access.

Despite its potential effectiveness, the acceptance of the platform by the HCPs in the Canadian Hospitals study differed depending on their professional background. Some HCPs were not entirely comfortable with the content of the questions and the information made available to them through using TickiT.

Interestingly, resident trainees, who are generally responsible for collecting this patient information on admission, universally accepted the tool. This population may be younger, and thus more comfortable with technology than the more senior HCPs, and compared to the more established senior HCPs, the resident trainees may be less rigid in their work practices. Low acceptance by the surgeons, who felt uncomfortable with the questions which formed the substantive backbone of the tool, is consistent with the low incidence of screening in this setting [12]. While the tool had many affordances and has the potential to meet the criteria outlined in the Triple Aim framework which evaluates innovations in the health care setting using 3 evaluation criteria: to improve the patient experience,. maintain or decrease costs, and improve care. These were not sufficient for overcoming some HCP’s discomfort with the content of questions.

The resistance shown by senior physicians to adopting this platform may be attributed to the challenges associated with changing the typical work practice in an established traditional workplace setting. In this setting the introduction of the platform could be considered eHealth disruptive technology [34], where technology initially disrupts the status quo but over time reconfigures clinical services. Increasing universal screening is providing a wealth of new information to a clinician and it may be threatening if he/she does not feel comfortable managing it, although the collection of such information may be considered standard of care. The introduction of a new technology such as ours in a clinical setting needs to be accompanied with strategies for supporting physicians in managing new health issues that arise from screening. In addition, as comfort with integrating information resulting from psychosocial screening into clinical encounters increases, additional evaluation will be required (eg, assessing how HCPs’ workflow changes in relation to the availability of psychosocial data, how length of appointments changes, etc). Future acceptance of the platform will depend in part on ensuring that there is a good fit between the content of the questions and HCP comfort in addressing the issues raised through the availability of that information.

The IHI has established a Triple Aim framework to evaluate changes in innovations in the health care setting. This framework uses three evaluation criteria to improve the patient experience, maintain or decrease costs, and improve care [35]. The Canadian Hospitals study [23] suggests that implementation of the platform has the potential to meet all these criteria. Our platform was universally accepted by the patients and provided an excellent patient experience. It was efficient and required less than 15 minutes to complete by the patient with no physician input, and had the potential to add value by improving compliance with recommendations for psychosocial screening of youth, and by contributing to low cost for obtaining data for research. Findings from the pilot study undertaken by Lam et al [23] suggest that psychosocial patient information can be collected from youth using the platform in considerably less time than a 30-minute face-to-face interview. In clinical settings where HCPs were unfamiliar with the patient, all HCPs obtained new information about their patients from the platform, highlighting the potential value of using TickiT to collect new patient information about sensitive issues. While this is a precursor for improved patient care, it does not guarantee improved care. Therefore, as the use of TickiT progresses from pilot applications into everyday clinical use, additional evaluations (eg, to determine whether or not physicians act on information gathered through TickiT, and to assess use through survey and/or interview of practitioners together with an analysis of pre-and post-use referral patterns) will have to be performed.

Given the variability in deliverey of health care from hospital to hospital and across clinical settings, an action-oriented approach to implementation [36] may be required in order to identify and address issues and challenges that arise as the TickiT platform is integrated into work practices in different clinical settings. In each setting where TickiT is introduced, decisions will need to be made about how the mobile device containing the questionnaire is administered, and which members of a care team receive and act upon results of the survey. Clinical adaptation requires planning, training, and support for HCPs to modify their practices.

Studies undertaken in the development of TickiT has been valuable in many respects. The co-creation model has led to numerous enhancements to the platform, and, since completion of the pilot studies summarized here, the basic platform design has expanded to accommodate new stakeholder requests (such as providing immediate feedback based on responses entered and links to health promotion resources). While co-creation processes have been successful and preliminary results from these studies have suggested that uptake and success of the platform are likely to continue, more widespread adoption may require more robust research to demonstrate the value of TickiT in meeting IHI Triple Aim evaluation criteria. Preliminary research has been invaluable in informing design of the platform and identifying issues (eg, discomfort among some HCPs with the content it yields) warranting further attention. In an environment where there is ample competition for health care dollars, evidence is required to demonstrate value. Undertaking additional research will increase the purchase appeal of TickiT and continue to contribute to product enhancements. Examples of such research could be aimed at addressing questions about costs of implementation and use of TickiT, changes in clinical practice which are proxy measures for improved health outcomes, and evaluation of research functionality of TickiT. Already the platform has been expanded to be available in any language, and the number of clinical scenarios for data collection has grown in the public health arena and specific sensitive clinical areas such as urology.

### Limitations

Pediatric outpatient clinical care is usually provided by a clinical team of HCPs, and allied health HCPs such as nurses often manage psychosocial issues. However due to the variety of clinical settings, there was considerable variability concerning how allied health were engaged in patient care from setting to setting. Hence they were omitted as participants from this study. Future studies would benefit from engaging all HCPs involved in the patient’s care.

Additionally, in the ambulatory care setting most of the visits were for follow-up care, and the clinical team already had a good understanding of the patient’s psychosocial status. Finally, the HCPs were not asked for detailed information regarding their experience with the platform design as this particular study focused on the feasibility of obtaining the information in clinical settings rather than on the means through which the content was received. Variations in response between study sites (eg, to the last questions) may reflect differences in how the application was introduced, time in waiting area, or other variables.

**Challenges in the Research Process**

While this paper has described two studies involved in the developmental process to create and evaluate an eHealth platform, there were a number of informal, iterative short feedback cycles performed with both youth and health care professional user groups that have not been reported, because they did not fall within the academic framework.

There are significant practical challenges of embedding the development of eHealth technology within a research environment. In an ideal world, there would be better mechanisms to support long-term research collaborations between industry and academic institutions. None of Canada’s research councils have programs to support independent assessment of software developed for health sector use. Additionally, industry timelines are inconsistent with academic timelines. For example, industry requires more rapid uptake of investigation, which is not feasible with the granting cycle process, and academic research is constrained by funding, duration, and a specifically described framework prior to commencement of the work that limits investigative exploration beyond the specific research project. The co-creation study provided the recommendations for and evaluation of early prototypes. Further development occurred outside the research environment to create a functional product. The feasibility study was conducted once the product met “industry standards” by an academic, impartial team. These choices were made by Shift Health Paradigms to uphold a priority of arms-length independent research evaluation, to garner the best evidence available within a reasonable timeframe.

### Conclusions

In this paper, we have described the early developments of the TickiT eHealth platform that was initially designed to engage the patient, enhance the relationship between patient and provider and improve efficiency. A Canadian Hospital pilot study suggested that TickiT was an effective and efficient means to perform psychosocial screenings of youth during health care encounters. The platform was exceptionally well received by patients and residents. However some HCPs appeared to be uncomfortable with the information obtained from the TickiT HEEADDSS questionnaire, highlighting the importance of considering both the content of information collected and the means of collection when introducing new technology. While eHealth strategies can enhance the quality of data collection and encourage new relationships between providers and patients, the technology alone will not suffice if the results do not align with the objective of the clinician, even if the technology promotes standard of care. Since completion of the pilot study, the basic platform design has expanded to accommodate new stakeholder requests, and new research teams have commenced further investigations with the platform in a variety of health settings. Further studies are indicated to determine cost effectiveness, utility, and implementation in other health care settings where patients face sensitive issues. The co-creative design approach addressed the needs of the various stakeholders envisioned as the first target users of TickiT and created a framework for development that can continue to leverage the powerful potential of eHealth technology in future development for a variety of clinical settings and scenarios.

## Multimedia Appendix 1

TickiT video.

## Multimedia Appendix 2

TickiT features.

## Abbreviations

ASQ: adolescent screening questionnaire
BCCH: British Columbia Children’s Hospital
CLAS: computerized lifestyle assessment scale
ECUAD: Emily Carr University of Art and Design
EHR: electronic health record
HCP: health care providers
HEEADDSS: home, education, eating, activities, drugs, depression, sexual health, safety
IHI: Institute of Healthcare Improvement
IT: information technology
PHI: personal health information
PIPEDA: personal information protection and electronic documents act
UI: user interface
