# Supplementary material for: Co-Creation With TickiT: Designing and Evaluating a Clinical eHealth Platform for Youth
Source: JMIR Res Protoc. 2013 Oct 18;2(2):e42. doi: 10.2196/resprot.2865 (PMC3806391; doi:10.2196/resprot.2865)
Supplement: Supplementary file 2 [file resprot_v2i2e42_app2.pdf]

TickiT for Health

Features Overview

Daniel Penn
daniel@tickitforhealth.com

Shift Health Paradigms© 2011
TickiT for Health™

TickiT

Here's an overview of TickiT's features in three domains:

Survey & Feedback Tool Features
page 3

Website Features
page 9

TickiT Services
page 13

Survey Features

Interactive Survey Templates

We’ve got lots of templates for you to choose from when developing your survey, from graphic buttons to sliders for scales, scrollers for numbers, and more.

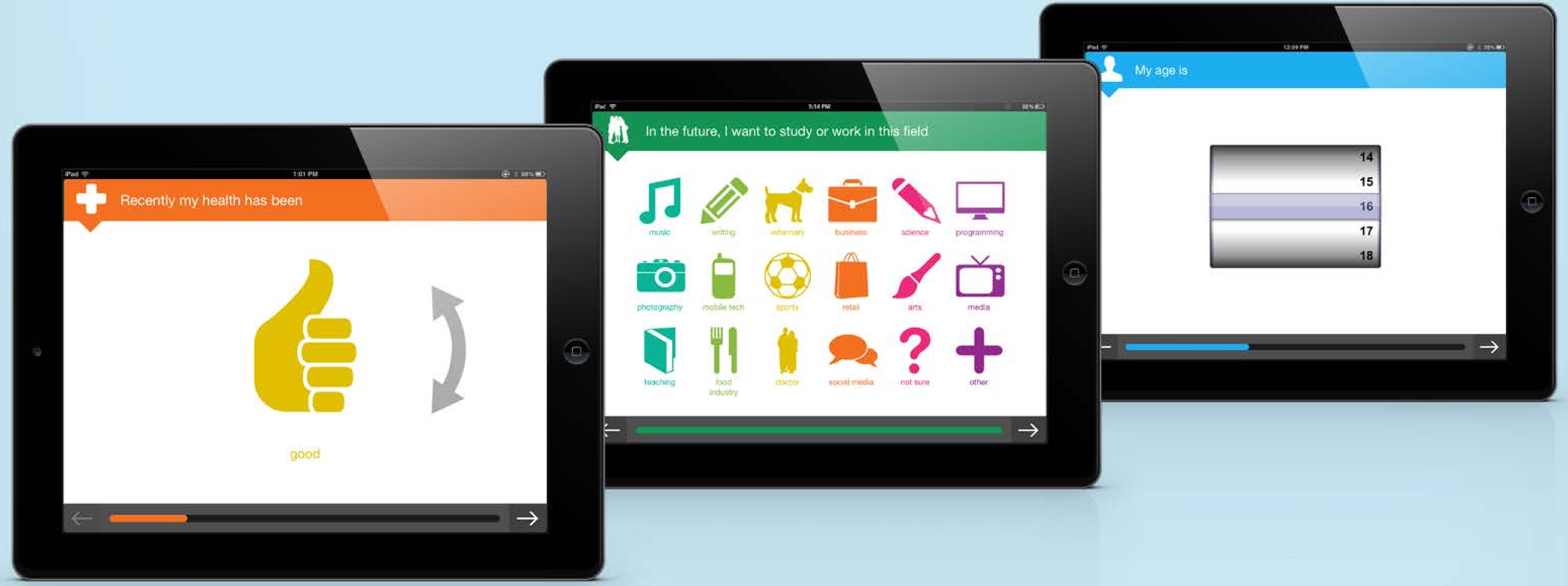

TickiT

Educational Patient Feedback

new feature!

After patients fill out your survey, our system dynamically provides them with educational feedback based on their responses. Provide stats, comparison graphs, and lifestyle suggestions to help educate your patients with tailored information.

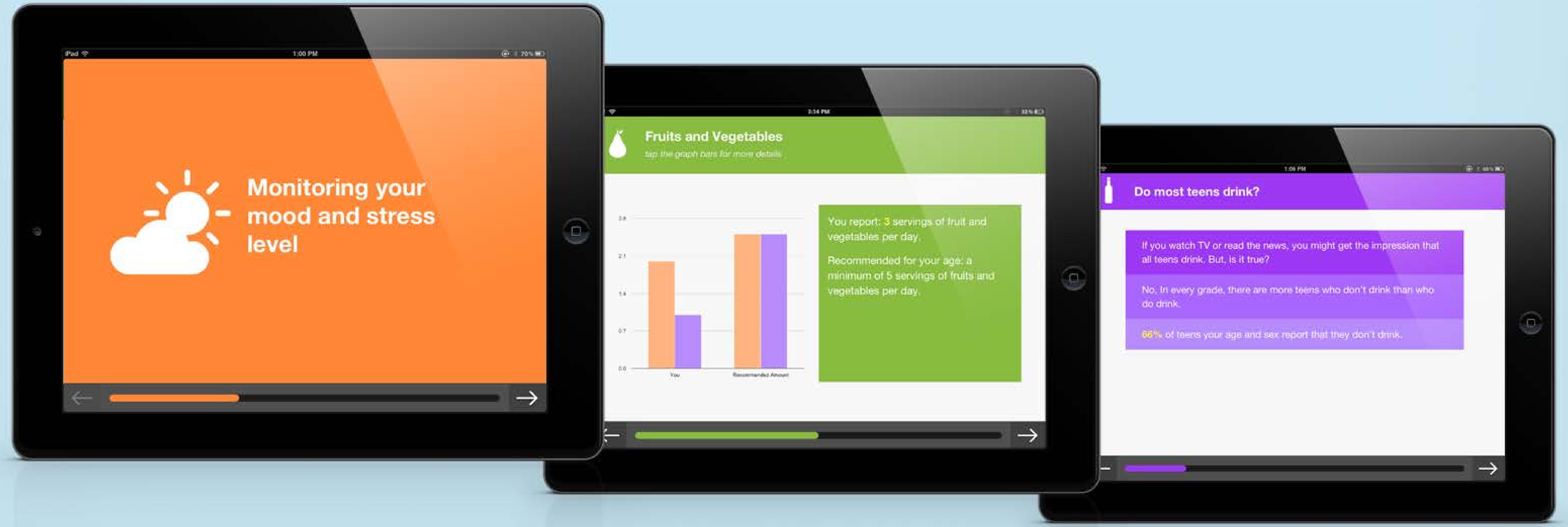

TickiT

Large Library of Icons

We’ve got over 200 graphic icons for your patients to engage with - and our library is constantly growing! TickiT gives you access to our entire library and can develop custom icons to meet your needs.

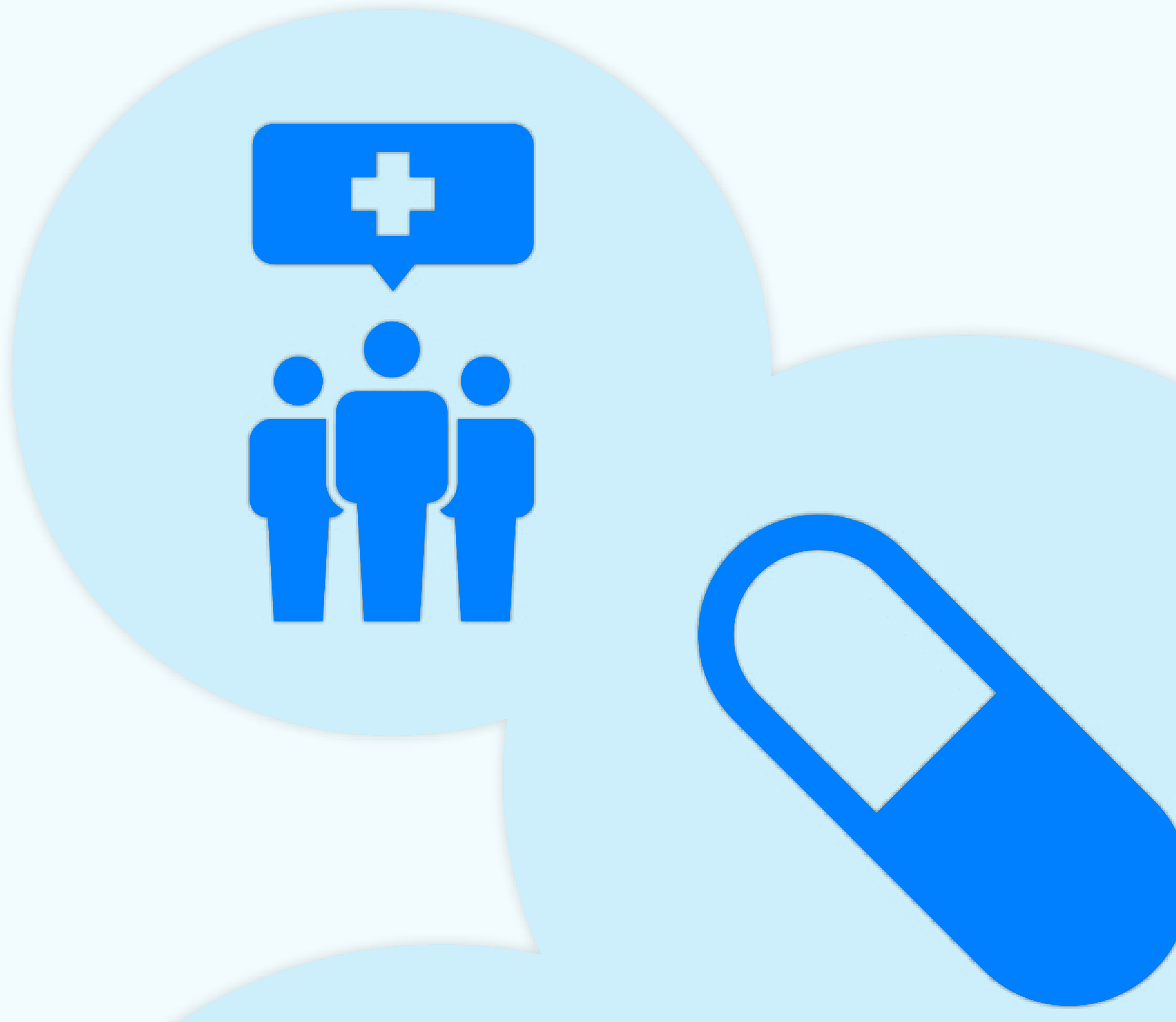

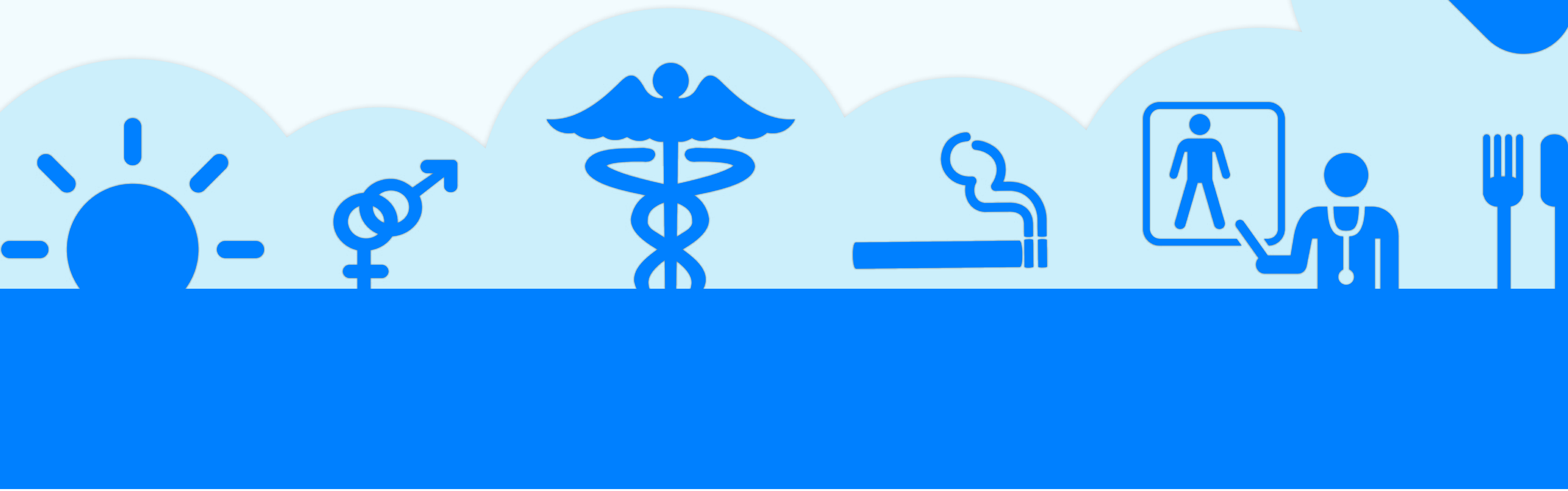

TickiT

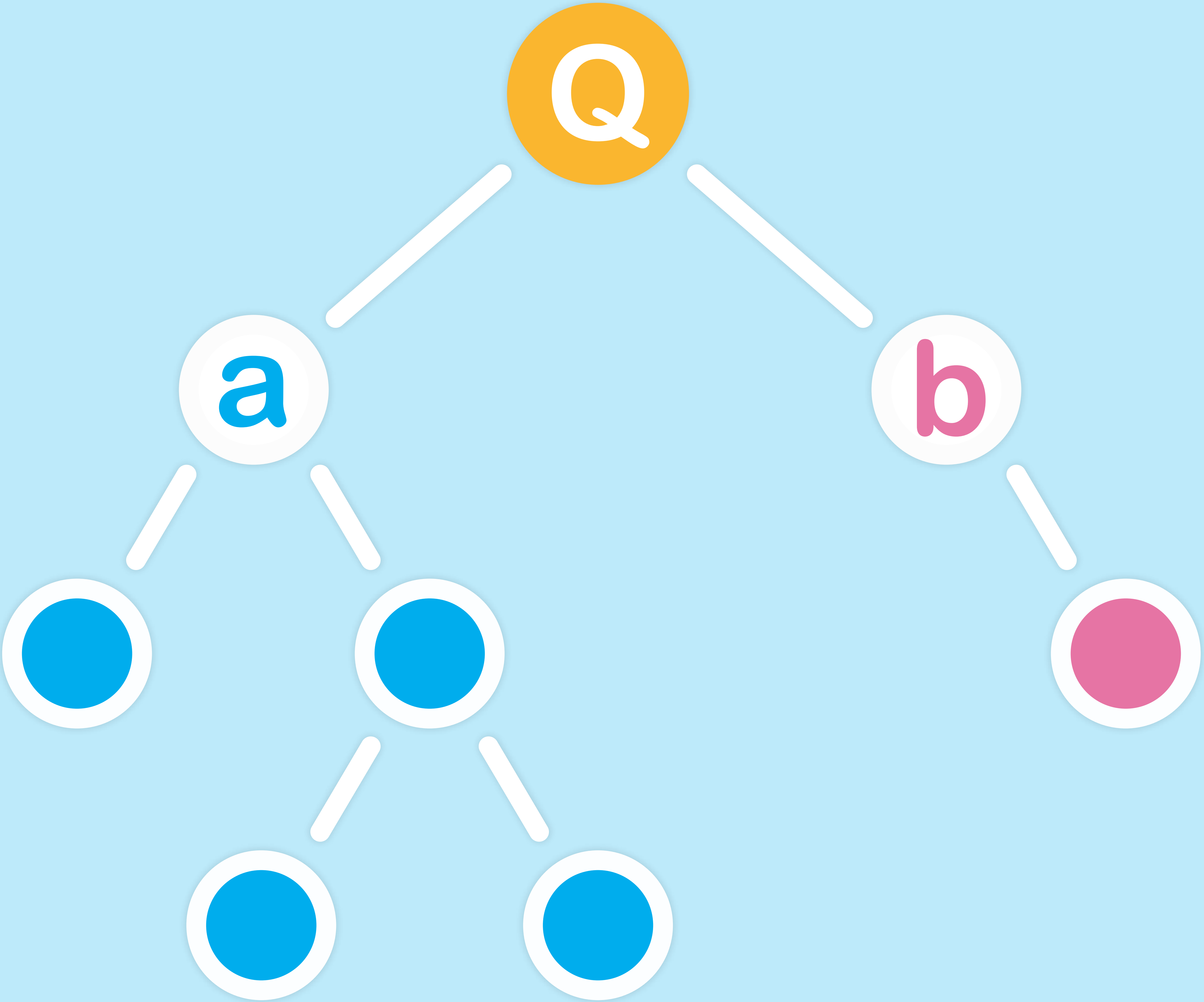

Branching To The Right Questions

Branching ensures patients only get asked relevant questions based on their previous answers. This can divide surveys by language, varying age groups, and specific topics.

TickiT

Multiple Language Support

Have a varied patient population? Ask questions in multiple languages and receive responses in English.

TickiT offers the HEADSS survey in French. We can also build your custom survey in multiple languages.

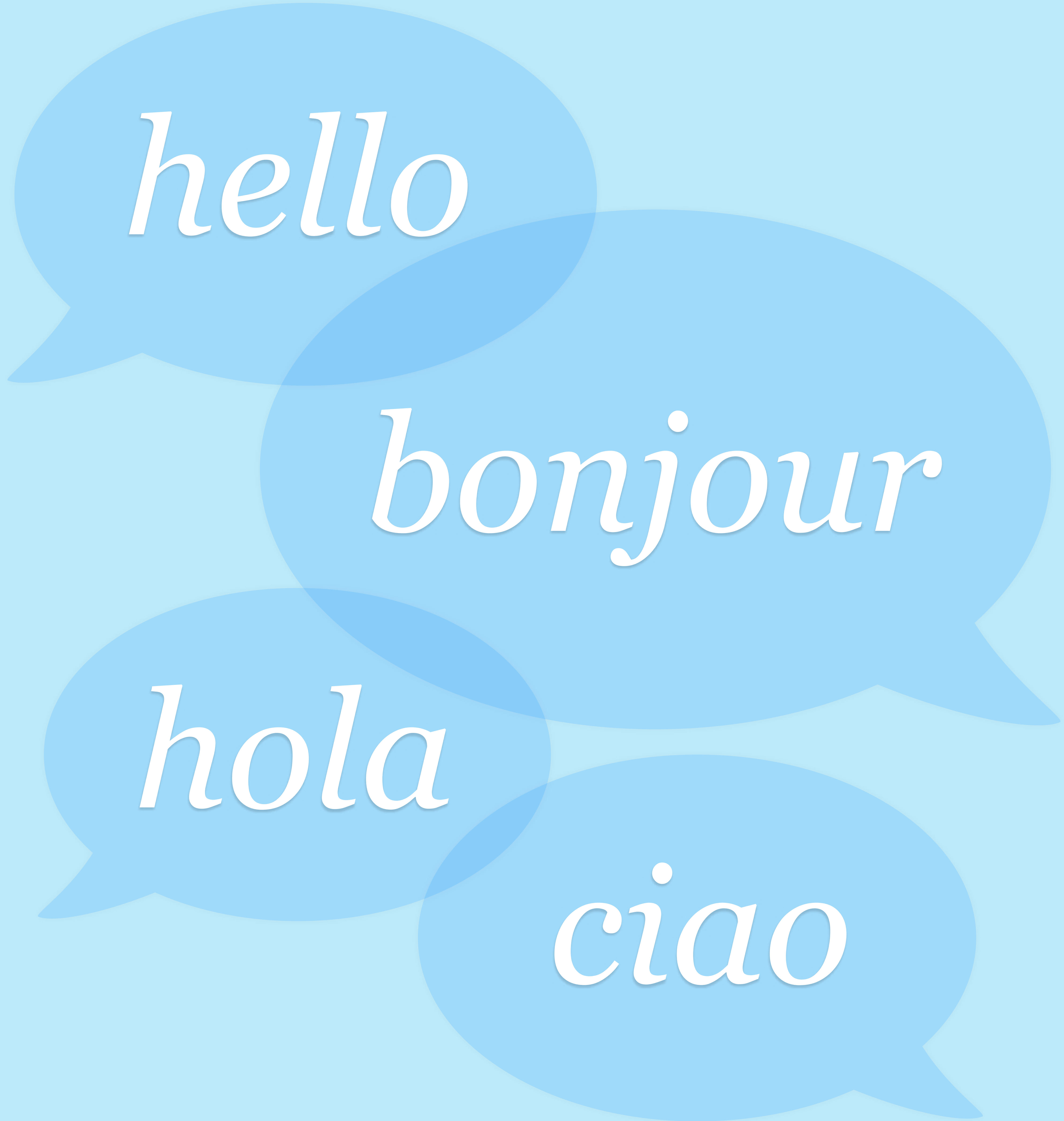

Website Features

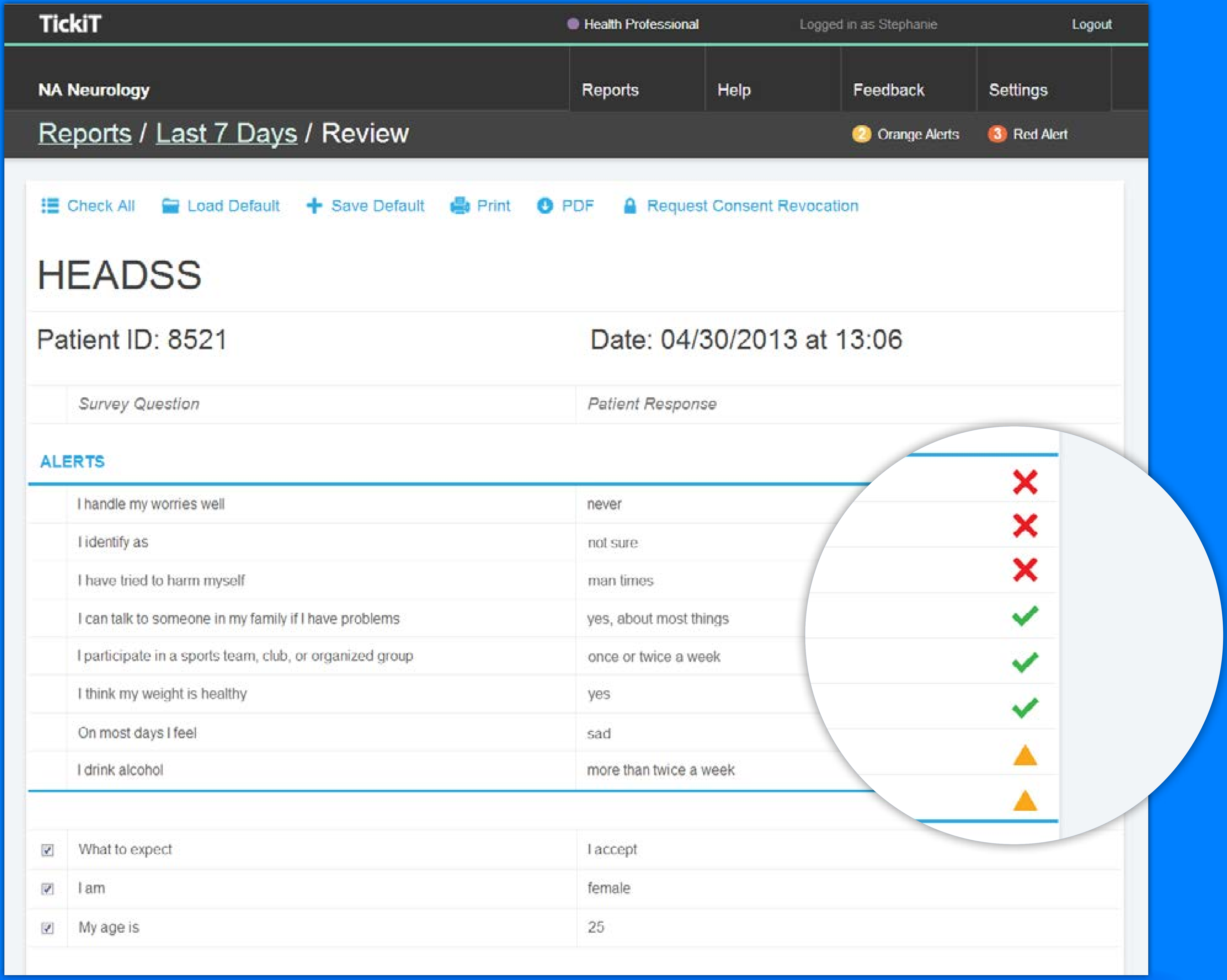

Report Alerts

Employing flags is an excellent way to quickly capture the most important information from your patient's response.

Catch red alerts, green protective factors and orange highlights right at the top of your surveys - our system highlights them automatically for you.

TickiT

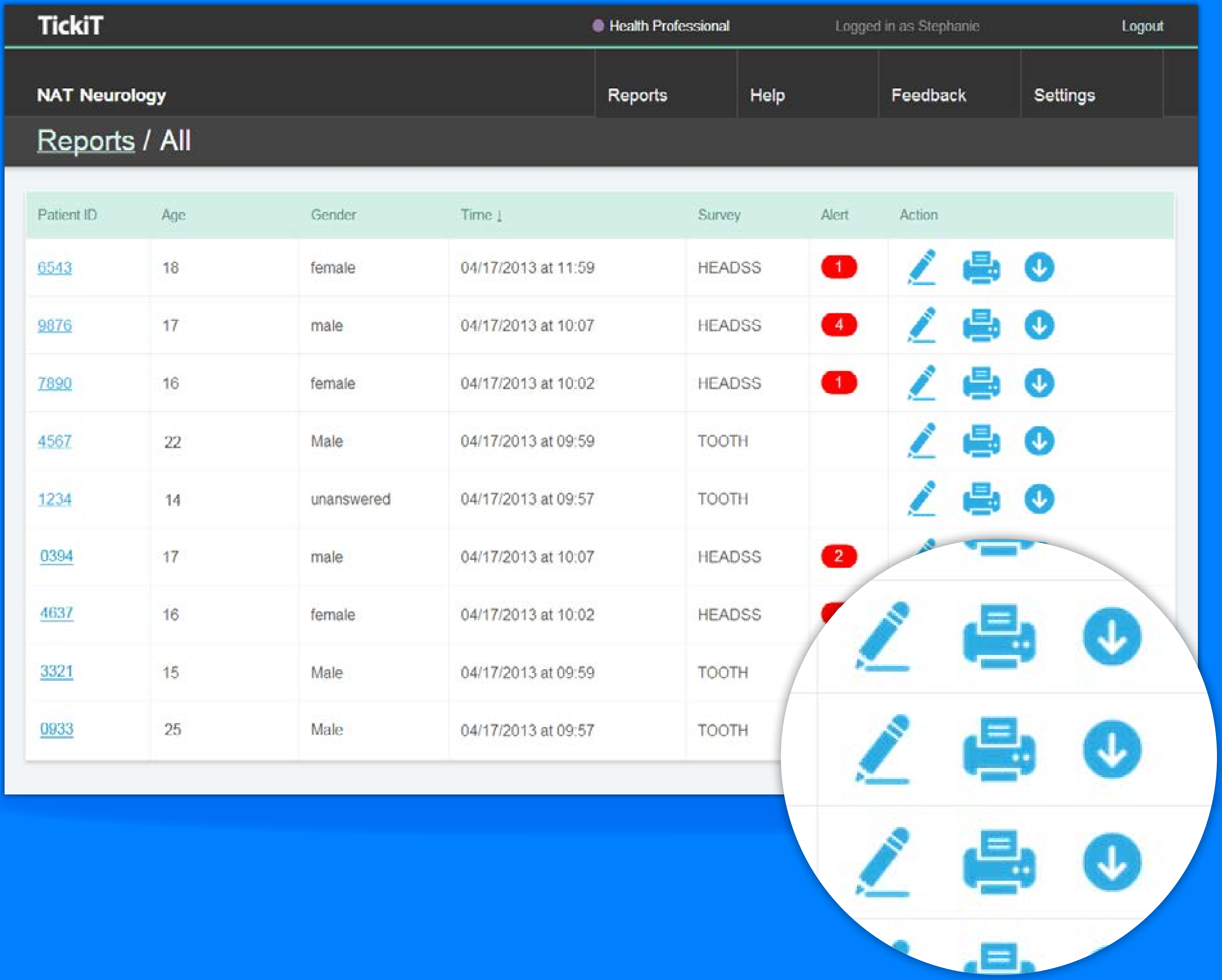

Reviewing Reports

TickiT offers various report views and export functions:

Website
Quickly review reports from your personal TickiT website.

PDF
Export PDFs to print for paper charts or export for EMR's.

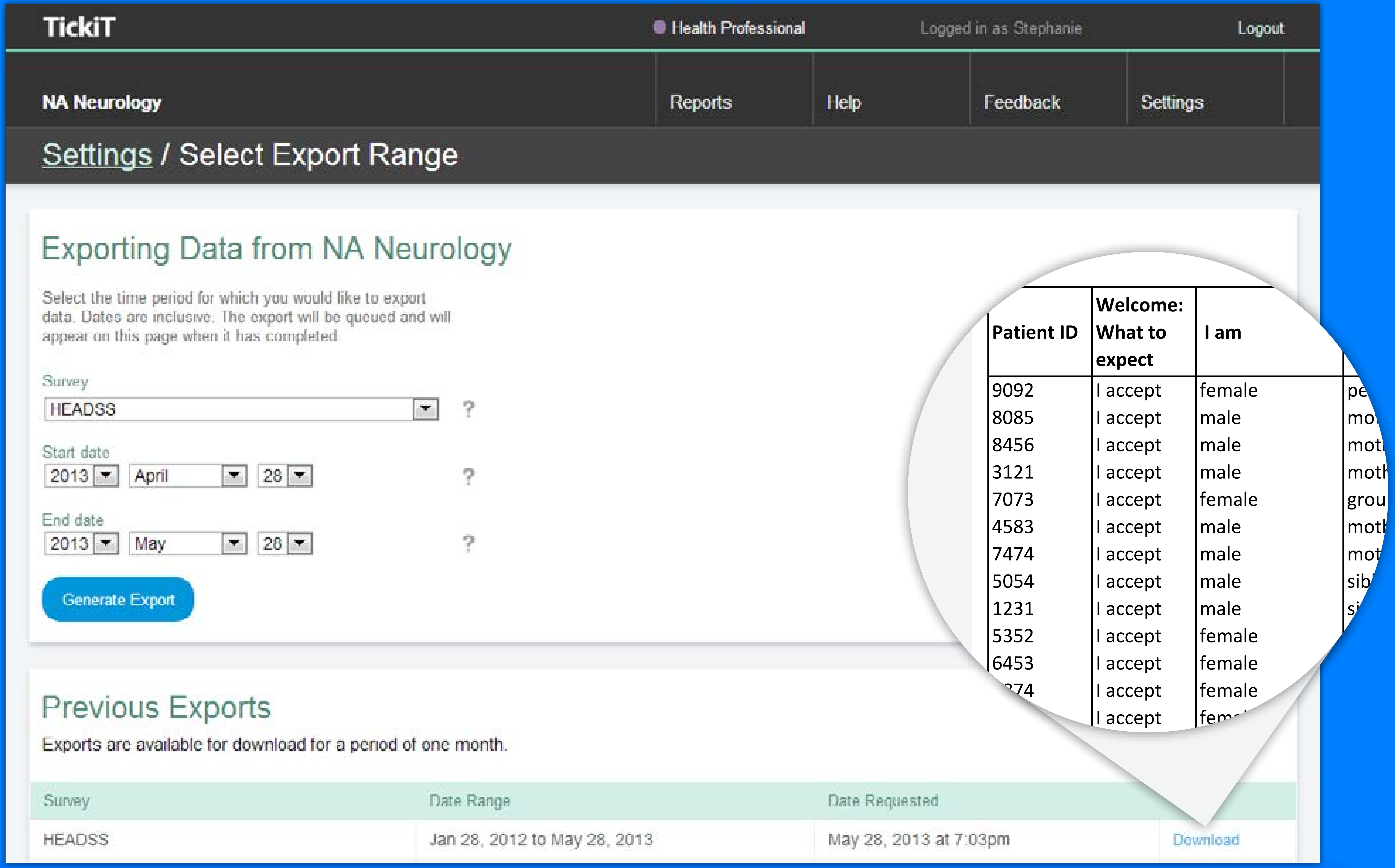

CSV Export for Research

This feature is a researchers' dream, export automatically aggregated reports between the dates of your choosing. No need for secondary data entry, with a couple of clicks you'll have all your data sorted for you in plain text or binary CSV formats.

TickiT Services

TickiT

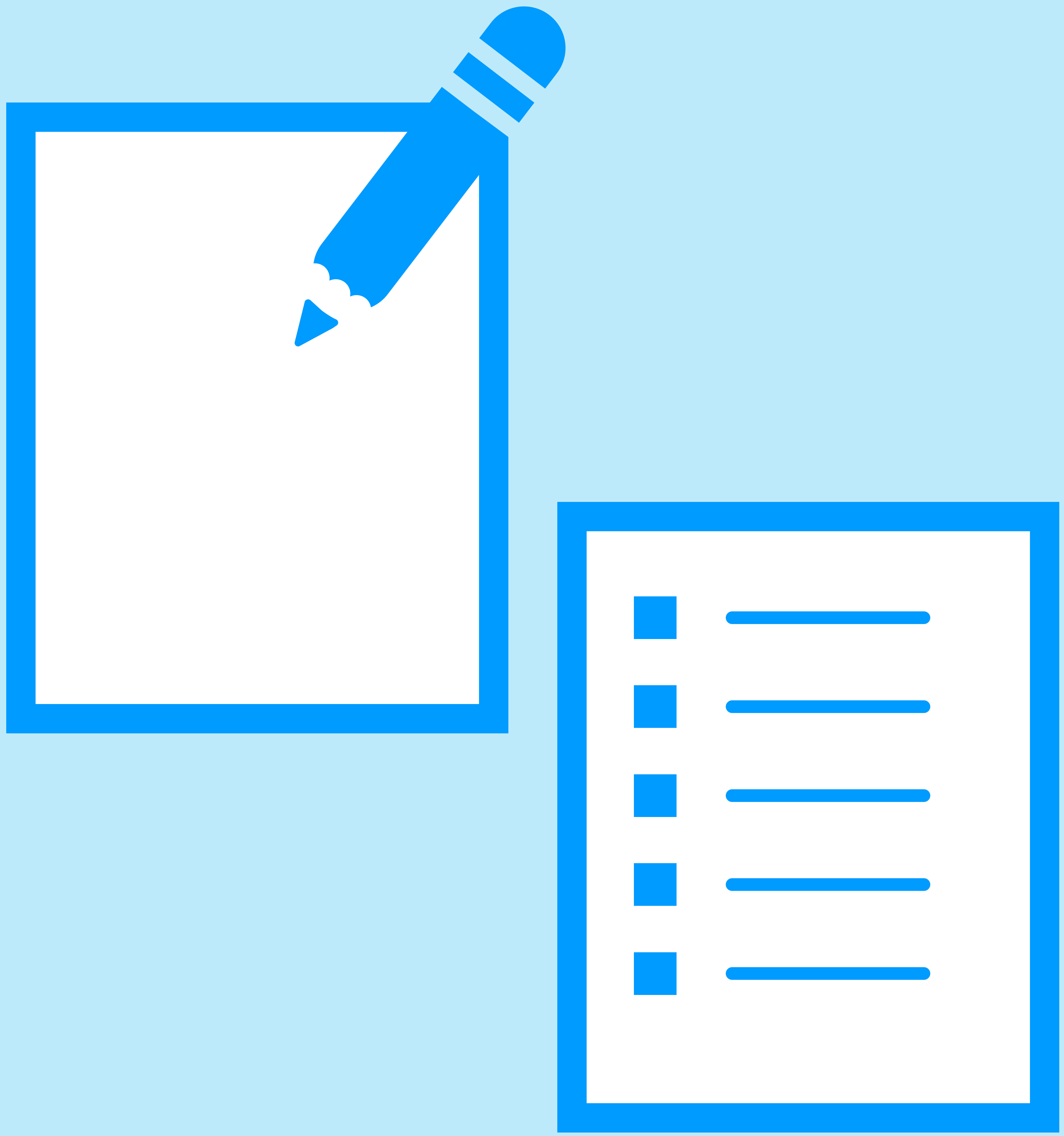

Create Custom Surveys or Choose Off the Rack

Custom Surveys

Have specific needs for your clinic or research project? We'll build you a custom survey with full interactivity. Our team also specializes in editing for low/wide literacy rates and patient friendly prose.

Validated Off the Rack Surveys

TickiT has a library of validated surveys written by other health professionals, simply sign up and get started collecting valuable patient data.

Security & Tech Specs

Security
We've done all our homework, with TickiT rest assured knowing your data is secure. We meet Canadian and American ePHI requirements and are HIPPA compliant.

Tech Specs
+ Available for iPad & Nexus 7 devices
+ iPads are available for lease, inquire for further details

TickiT Security Specs

TickiT meets eHealth standards including:

A. Encrypted data transmission
B. Password protected data
C. Secure data storage on dedicated servers
D. Secure backup
E. Patient consent
F. Unlimited users with role based access
G. End user agreement
H. Audit log for monitoring
I. Limited patient identifiers: no name or DOB on the questionnaire
J. Company Privacy Impact Assessment available on request

TickiT

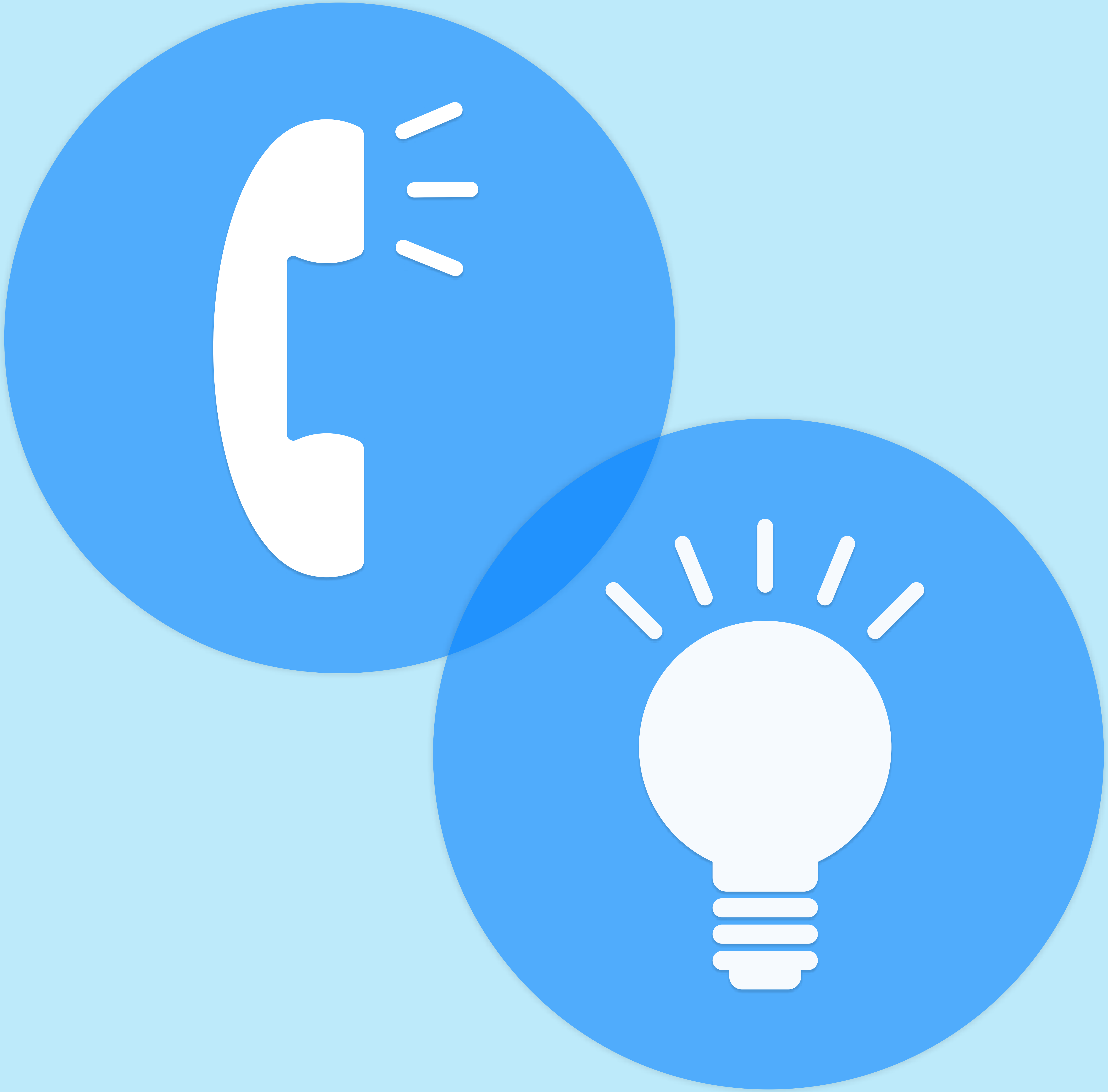

Unlimited Tech Support & Suggestions Box

As a TickiT customer your opinion matters, if you have an idea for a useful new feature, or think something could be improved to aid your workflow - let us know! Expect follow up calls and the chance to share your thoughts.

Contact us to discuss how TickiT can work for you.

info@tickitforhealth.com | www.tickitforhealth.com

TickiT
